# Notifications and Health Consequences of Unauthorized Pharmaceuticals in Food Supplements

**DOI:** 10.3390/pharmacy11050154

**Published:** 2023-09-23

**Authors:** Maja Amidžić, Jelena Banović Fuentes, Jovica Banović, Ljilja Torović

**Affiliations:** 1 Department of Pharmacy, Faculty of Medicine, University of Novi Sad, Hajduk Veljkova 3, 21000 Novi Sad, Serbia; 988d15@mf.uns.ac.rs (M.A.); 990d15@mf.uns.ac.rs (J.B.F.); 2 Department of Internal Medicine, Faculty of Medicine, University of Novi Sad, Hajduk Veljkova 3, 21000 Novi Sad, Serbia; 987d15@mf.uns.ac.rs; 3 Center for Medical and Pharmaceutical Investigations and Quality Control, Faculty of Medicine, University of Novi Sad, Hajduk Veljkova 3, 21000 Novi Sad, Serbia

**Keywords:** food supplements, pharmaceuticals, adulteration, health risk

## Abstract

Health concerns associated with the consumption of food supplements rise in parallel with the rise in the popularity and market availability of these products. In pursuit of data related to the unauthorized presence of pharmaceuticals in food supplements, the Rapid Alert System for Food and Feed (RASFF) database was searched for the 2011–2022 period. The most “popular” pharmaceuticals for the adulteration of food supplements were phosphodiesterase-5 inhibitors (235 records); anorexics and laxatives (76), including sibutramine and its active metabolite N-didesmethyl sibutramine, phenolphthalein and 2,4-dinitrophenol; stimulants, among which 1,3-dimethylamine (97), and synephrine (53) were the most numerous; nootropic drugs (24); anabolics and prohormones (16); and cannabinoid cannabidiol (14) (pending authorization as a novel food ingredient). Over 65% of notifications of interest were classified as serious risks, and over 80% of these were alert or border rejection notifications, mainly generated as a result of official control on the market. The alarming number of RASFF notifications should be considered a public health issue, demanding clear and targeted recommendation for action for the legislature and authorities. A harmonized nutrivigilance system should be considered as a tool to detect and scrutinize the adverse health effects of food supplements, along with measures to improve their safety, quality, and testing.

## 1. Introduction

Food supplements are defined as “concentrated nutrient sources or other substances having a nutritional or physiological impact, that are used alone or in combination to supplement a regular diet” [1]. The physiological effects of food supplements have not changed their classification as foods rather than medications. This makes it more difficult to identify adulterations, since it suggests that food supplements are not subjected to the monitoring, reporting, evaluation, and action to adverse reactions in the same way that medications are [2]. The safety of the product is the responsibility of the manufacturers, who must commit to Good Manufacturing Practice (GMP) compliance, through the control of the production process and verification that products meet their established quality specifications and limits on contaminants (chemical, biological, radiological, or physical elements which have potential to cause health risks, depending on the level of exposure) [3]. However, adulteration encompasses contamination, implying the intentional addition of extraneous ingredients/substances to a food, for economic (and technical) benefits.

In recent years, consumers’ interest in the potential of food supplements to support their health has continued to rise. According to research conducted by Ipsos for the Council for Responsible Nutrition and European Public Affairs, more than three-quarters of Americans and almost 90% of Europeans report using nutritional or food supplements, and most supplement users continue to take them regularly. Such findings support a commonly held belief that food supplements are healthier and less hazardous than synthetic medications. An investigation of public perception showed that as many as 84% (USA) and 69% (EU) of consumers trust the food supplement industry [4,5], which is not surprising given that food supplement manufacturers are only obligated to report serious adverse events [6]. It has been estimated that the growth of the global market of food supplements will reach a value of 128.64 billion by 2028 compared to 71.81 billion in 2021, and the value of the European market will be over 33 billion [7]. However, it has to be emphasized that the prospective assessment of food supplements for quality manufacturing, efficacy and safety is not in the provisions of either EU or USA laws.

A recent review of the regulatory landscape of food supplements and herbal medicines from a global perspective has summarized a longitudinal comparison of their status among the different national jurisdictions; despite certain inconsistencies (e.g., classification as medicines or supplements), the one commonality is that the prime focus is the safety of consumer [8]. Within the EU, food supplements are regulated under the general food law [9]. There is no centralized pre-market authorization; some countries have an established pre-market notification system, but with no common pre-market placement rules. The US Food and Drug Administration (FDA) regulates finished food supplement products and proper labeling under the Dietary Supplement Health and Education Act (DSHEA) [10], relying on post-market surveillance, while the Canadian system includes both pre- and post-marketing surveillance [11]. Post-market surveillance programs are reliable resources but are limited to removing unsafe products from the market.

Risks related to food supplements include intrinsic toxicity, accidental contamination, interactions with other supplements or foods/drugs, and intentional adulteration [12]. Some producers boost the effects of their products through the addition of illegal pharmaceuticals. With this strategy, they fulfill the initial expectations of the consumers by creating an effective supplement out of an ineffective one. It is not reasonable to anticipate instantaneous results from food supplements if their purpose is to offer preventative and supportive care for one’s health [13]. Supplements adulterated with pharmaceuticals and/or their synthetic derivatives are a major problem that might seriously compromise consumers’ safety, especially those containing prescription drugs, where a number of cases of serious adverse events and even death cases were recorded [14].

Existing surveillance systems implemented in various countries provide information on the adverse effects of herbal products, e.g., the ADR reporting system in China, the MedWatch program (FDA) in the US, Medsafe in New Zealand, and Pharmacovigilance (EMA) in the EU [8]. In the US, the Center for Food Safety and Applied Nutrition (CFSAN) Adverse Event Reporting System (CAERS) database was launched by the FDA to support the post-market monitoring and surveillance of adverse effects associated with food and food supplements, as well as cosmetics. It contains data voluntarily submitted by government agencies, healthcare professionals and consumers, and mandatory reports of post-marketing serious adverse effects from food supplements submitted through the MedWatch program [15]. Although European Union legislation does not include a provision to establish a nutrivigilance system for the rapid identification of possible harmful effects associated with the consumption of food supplements/fortified foods/novel foods/foods for specific population categories, several national schemes are in operation. Reporters are healthcare professionals such as doctors, pharmacists, and dieticians, who observe or become aware of adverse effects related to the consumption of these foods, but also manufacturers and consumers. The main short-comings are the non-mandatory status of reporting and under-reporting, lack of harmonization of the data collection, and different methods for the assessment of severity and causality. A European-harmonized nutrivigilance system would allow a common understanding and to facilitate information exchange, leading to an increase in the number of reports, early risk detection, strengthening of health risk assessment, and improvement of public health [12]. Existing European systems for reporting food supplements’ safety include the International Nutrivigilance network (driven by France), the Rapid Alert System for Feed and Food (RASFF), and the Emerging Risks Exchange Network (EREN, run by the European Food Safety Authority (EFSA)). These systems aim to protect consumers through early warnings, enabling the rapid implementation of management measures when needed, based on the risk assessment. It has to be emphasized that RASFF is not suitable for reporting food supplements’ adverse effects.

The RASFF is a well-known example among the food alert systems operating worldwide [16]. The RASFF was set up as a reliable mechanism for sharing data between authorities of the EU member states, which are required by law to provide data on any direct or indirect health risks associated with food and feed. It has been in place since 1979 and has become an integral public health protection strategy [17]. It was based on Article 50 of Regulation (EU) 178/2002 [9], operates around the clock, provides high-quality metadata, and makes summaries of recently sent RASFF alerts available to the public via an online database that can be searched and navigated interactively [9]. The RASFF system has evolved and been upgraded over the years to become the comprehensive database of food safety risks that it is today [16,18]. The types of RASFF notifications are alerts, information (either for attention or for follow up), and border rejections. Alert notifications are submitted when product presenting a serious direct or indirect health risk is on the market and rapid action is required; the information notification relates to products in which the risk has been identified, but the nature of the risk does not require prompt action or the product is not yet/no longer on the market; border rejection notifications are reported if consignments are rejected at the EU’s external border [19]. The “serious” risk decision was introduced in 2011 for border rejection and in 2012 for alert notifications. 

Numerous studies were based on data extracted from the RASFF portal. Regarding food supplement fraud, a study based on both European data obtained from the RASFF and Polish national data indicated that most irregularities reported were related to mislabeling the final product’s composition and attributed properties related to nutrition and/or health claims [20]. Several studies dealt with the occurrence of pharmaceuticals causing specific physiological effects such as weight loss [21,22] and sport and sexual performance enhancement [22] or with substances used both in supplements and medicines [23]. On the other hand, some research groups conducted analytical investigations of unauthorized pharmaceuticals in food supplements. Liquid chromatography coupled with the high-resolution mass spectrometry screening of 124 forbidden substances belonging to 13 classes of pharmaceuticals applied to 110 food supplements collected from the internet market or during official controls in Italy revealed that 4.5% were non-compliant [24]. High-performance liquid chromatography coupled with mass spectrometry applied on weight loss supplements offered for sale in the United Arab Emirates revealed that 15.3% of the samples contained sibutramine, 13.9% contained phenolphthalein, and 5.1% contained fluoxetine [25]. 

The rationale for the current work was to find out whether there is a problem related to unauthorized pharmaceuticals and their synthetic derivatives in food supplements on the EU market, as reported through the RASFF portal, and, if so, the scale of it; to obtain an insight into existing trends in the adulteration of food supplements in terms of groups of pharmaceuticals and individual compounds most frequently found; and to provide an overview of their undesired pharmacological effects and consequent health risks.

## 2. Materials and Methods

The search of the online RASFF database was conducted for product category—“*dietetic food, food supplement and fortified food*” linked to hazard category—“*composition*”, taking into consideration notification type (alert, border rejection, or information) and risk decision (serious or not, or undecided), as well as notification basis and action taken, within the time frame of 1 January 2011 to 31 December 2022. The data were exported to Excel and refined manually as a way to exclude products other than food supplements and adulterated raw materials intended for further processing, as well as herbal teas, food intended for specific diets, energy drinks, baby formulae, and substances offered for online sale without data indicating that it is a food supplement. Notifications were grouped based on individual compounds belonging to selected groups of pharmaceuticals, verified and counted. Notifications to which a “serious risk” decision was assigned in RASFF database were extracted and divided based on notification type. Notification types “alert” and “border rejection” were further linked with notification bases and actions taken. It is important to note that unauthorized pharmaceuticals have to be identified through laboratory testing as they are not on the product label. However, more detailed information related to the methods used to verify the presence of unlabeled pharmaceuticals in the supplements was not available on RASFF portal.

## 3. Results and Discussion

### 3.1. RASFF Notifications Related to Unathorized Pharmaceuticals in Food Supplements

**Figure 1:**
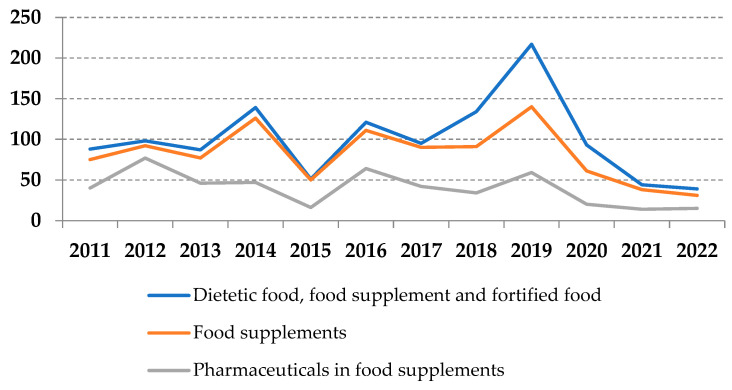
Distribution of notifications (RASFF database, 2011–2022).

The automatic search of RASFF database notifications related to the composition of food category “dietetic food, food supplement and fortified food” over the specified 12-year period showed great variations in the annual distribution of records, from 39 in 2022 to 217 in 2019, making a total of 1206 (Figure 1). When food supplements were singled out, the number of notifications was 982 (81.4%), varying from 31 in 2022 to 114 in 2019. Usually, supplements were adulterated with unauthorized substances, unauthorized ingredients, or unauthorized novel food ingredients or legal substances, but present in doses significantly higher than allowed. It is important to note that as many as 324 (32.9%) notifications were related to multiple instances of non-compliance, in the sense of the simultaneous presence of several unauthorized compounds (there were cases where compounds were of the same class but also multiple classes were found together) and that is the reason why the number of revealed records is significantly higher than the total number of notifications in the RASFF database.

**Figure 2:**
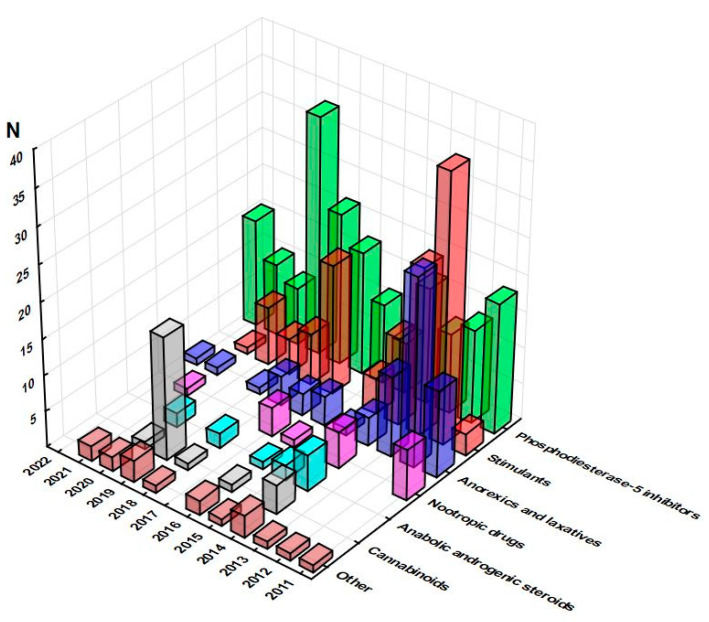
Dynamics of notifications related to specific groups of pharmaceuticals over the years (RASFF database, 2011–2022).

Out of 982 notifications related to the non-compliance of food supplements composition, 474 (48.3%) were related to pharmaceuticals identified as adulterants, most of which (77) were recorded in 2012, as opposed to the minimum (14) observed in 2021. These pharmaceuticals could be divided into the following groups: phosphodiesterase-5 inhibitors (PDE-5i), anorexics and laxatives, stimulants, nootropic drugs, anabolic and prohormones, cannabinoids, and others, including antibiotics, antioxidants, and hypnotics. The dynamics of notifications related to specific groups of pharmaceuticals over the years are presented in Figure 2, showing the constant presence of phosphodiesterase-5 inhibitors, stimulants and anorexics and laxatives over the years, despite rather uneven distribution of notifications.

**Figure 3:**
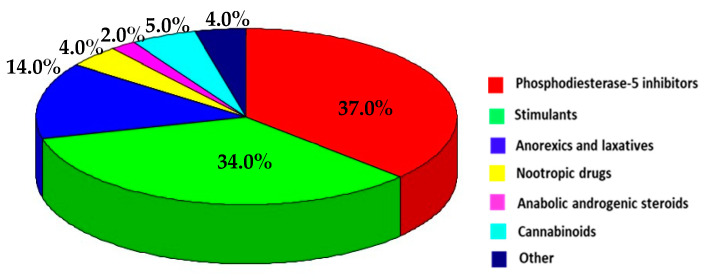
Distribution of RASFF records related to unauthorized pharmaceuticals in food supplements across the groups of pharmaceuticals (RASFF database, 2011–2022).

A total of 10 out of 474 notifications were related to supplements adulterated with more than one pharmaceutical, as follows: stimulants and AAS (3); PDE-5i and anorexics and laxatives (2); stimulants and unclassified pharmaceuticals (2); AAS and cannabinoids (1); PDE-5i and unclassified pharmaceuticals (1), and nootropic drugs and unclassified pharmaceuticals (1). With the aim of interpreting the data as reliably as possible, as well avoiding the assumption of which of the pharmaceuticals was responsible for the risk decision and notification type, the number of notifications for each pharmaceutical group was increased as follows: three for PDE-5i, two for anorexics and laxatives, five for stimulants, one for nootropic drugs, four for AAS, one for cannabinoids, and four for the group of unclassified pharmaceuticals (Figure 3).

The classification of RASFF notifications is based on the severity of the health risk and the response time required to eliminate the risk. Alert and border rejection notifications are initiated when any RASFF member identifies a counterfeit product posing a serious health risk on the market or during import control, with the intention of informing the other members of the identified risk, allowing them to take the necessary precautions, and preventing the re-entry of such products into the EU [26]. Altogether, 65.5% of all notifications related to unauthorized pharmaceuticals in food supplements were classified as a serious risk, 80.1% of which were serious alerts and serious border rejections (60 notifications were of information type). Regarding the risk decision, it should be noted that majority of those “undecided” were from the period prior to the introduction of the “serious” risk decision in the RASFF system in 2011/2012 (e.g., 24 of 28 such notifications were related to PDE-i).

**Figure 4:**
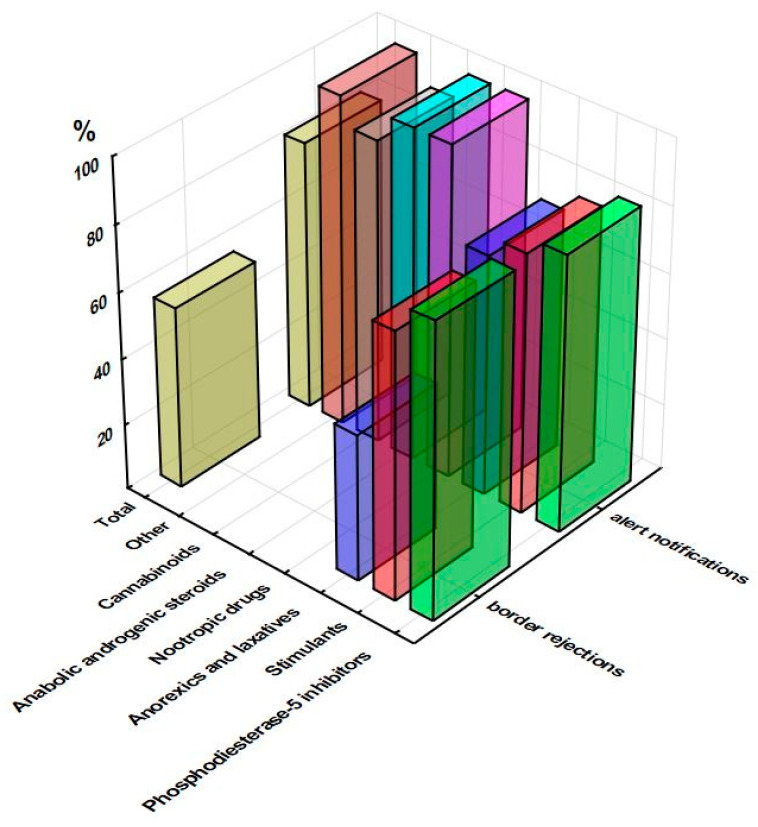
Percentage of serious alerts and serious border rejections in total notifications related to a group of unauthorized pharmaceuticals in food supplements (RASFF database, 2011–2022).

The substantially lower total number of serious border rejections in comparison with the total number of serious alerts (71 vs. 261) was a result of the predominance of alerts in all groups of pharmaceuticals (e.g., 18.6- and 7.8-fold in case of stimulants and PDE-i, respectively) except anorexic and laxatives (2.4-fold difference). It should be noted that border rejection notifications have a limit in terms of not specifying the consignment’s capacity, so it remains unclear how many supplements were brought into question. To avoid the repetition of data, Figure 4 shows the percentage of serious alerts and serious border rejections in total notifications related to a specific group of pharmaceuticals; while the involvement of individual unauthorized substances will be further described.

**Figure 5:**
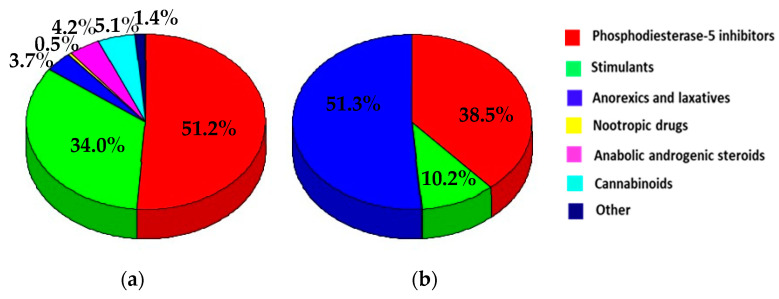
Percentage contributions of the groups of pharmaceuticals to the total number of (**a**) serious alerts and (**b**) serious border rejection notifications related to unauthorized pharmaceuticals in food supplements (RASFF database, 2011–2022).

The percentage contribution of a group of pharmaceuticals to the total number of serious alerts/serious border rejection notifications related to all groups of pharmaceuticals taken together is presented in Figure 5a,b, respectively. Phosphodiesterase-5 inhibitors led with slightly more than half of the total number of serious alerts (51.4%), followed by stimulants (34.1%), while other groups contributed with up to 5.1%. Anorexics and laxatives were responsible for 51.3% of serious border rejections, and phosphodiesterase-5 inhibitors for 38.5%.

**Figure 6:**
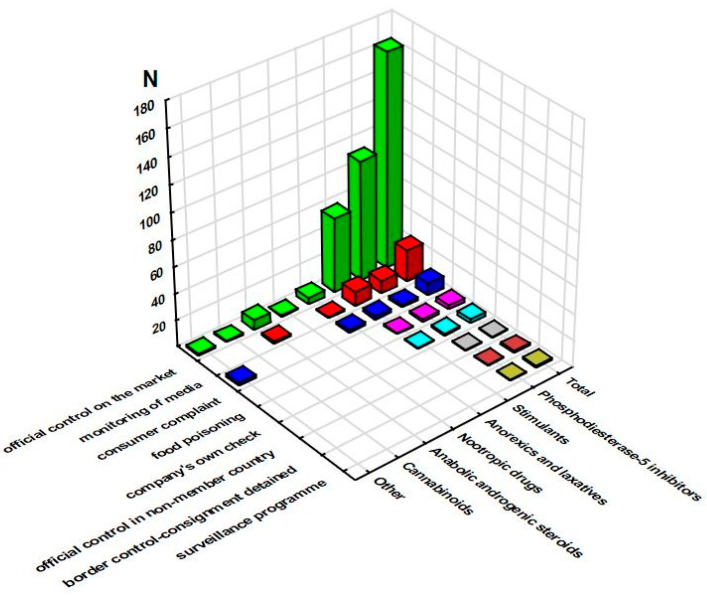
Sum of notification bases for serious alert notifications across the groups of pharmaceuticals (RASFF database, 2011–2022).

**Figure 7:**
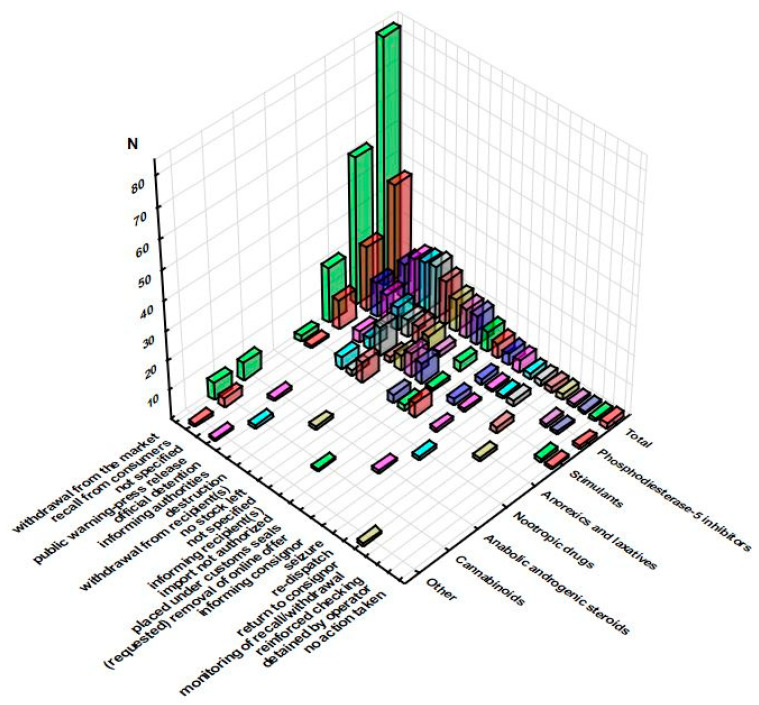
Sum of actions taken for serious alert and serious border rejection notifications across the groups of pharmaceuticals (RASFF database, 2011–2022).

The number of notifications, their type, and the assigned risk decision were further complemented with the data related to the notification bases (Figure 6 shows alerts, as all border rejections were based on border control—consignment detained or under custom) and actions taken (Figure 7).

Regarding notification basis, 80.5% of overall serious alert notifications were generated through the official control on the market, indicating that this measure has a great influence on the preservation of public health and effort should be made toward its intensifying. The groups of pharmaceuticals involved in serious alert notifications resulting from the official control on the market were the following: PDE-5i, 52.6%; stimulants, 32.9%; cannabinoids, 6.4%; and AAS, 4%. The monitoring of media was less represented notification basis, but it showed a similar distribution among the mentioned groups of pharmaceuticals, with PDE-5i (41.7%) and stimulants (45.8%) being the most numerous. This means that the media have great potential, and it is necessary to additionally connect the regulatory authorities with the media in order to improve the flow of information. The worrying fact was that in nine cases the basis for notification was a consumer complaint, and in three even food poisoning, which means that here the prevention did not lead to results, adulterated supplement reached consumers, and the damage was already done.

The number of items of data on action taken could be greater than the sum of notifications (as in case of PDE-i), since notifications from 2022 contain more outcomes for the same supplement. Although 58% of serious alert and border rejection notifications resulted in either the withdrawal of the questionable product from the market (31.7%), recall from consumers (14.1%), official detention (6.5%), or its destruction (5.7%), the practice of adulteration will continue, because, despite the financial losses caused by such actions, for an irresponsible manufacturer, profit could justify the stated losses. The losses could include not only cost of the supplements but also financial penalties for serious legal ramifications, but the RASFF does not provide any information on further steps taken towards manufacturers in response to violations. Public warning press release was linked to only 6.1% of all serious notifications, making this action underestimated due to the great influence of the media on public opinion, as well as the availability of information. The online market has made supplements more available than ever, but appropriate control mechanisms are still underdeveloped and hard to implement. The removal of the product from the online market could be requested, but it is hard to know whether that request has been truly fulfilled. Based on a survey conducted on the analysis of the RASFF data in the 2003–2016 period, Polish researchers have concluded that, although the majority of EU member states (MS)’ authorities consider drug-adulterated food supplements to be a public health threat, the responsibilities of the official authorities concerning such products do not appear to be clearly defined, and stricter legislative solutions are needed [27].

**Figure 8:**
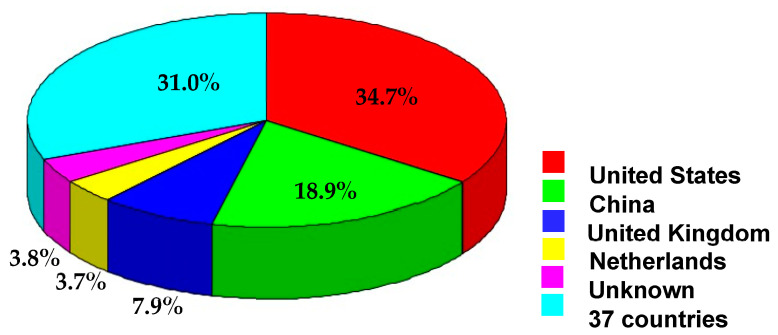
Countries of origin of food supplements notified to contain unauthorized pharmaceuticals (RASFF database, 2011–2022).

Data related to the origin countries indicate the countries that have more intense export or exhibit recurrent notifications, and therefore should be of particular concern. It was observed that most of the hazardous products were from the USA and China (Figure 8). Among European countries, the top-positioned were the United Kingdom (7.9%); the Netherlands (3.7%); Poland and Latvia (1.9% each); and Hungary, Bulgaria, and Austria (1.7% each).

### 3.2. Pharmaceuticals Identified in Food Supplements

Individual pharmaceuticals identified in food supplements, including their trade-names and indications for use, are presented in Table 1, together with their report numbers, while Table 2 shows a summary of the determined concentrations of pharmaceuticals in food supplements.

#### 3.2.1. Phosphodiesterase-5 Inhibitors

Many men, burdened by traditional beliefs, hide their erectile dysfunction, so it goes unnoticed. For this reason, unaware of the potential dangers to their health, some of them turn to widely accessible food supplements that are marketed as being entirely natural and safe, but in reality contain phosphodiesterase-5 inhibitors.

PDE-5i rank first among the pharmaceuticals illegally added to food supplements (Figure 3). They were recorded in 180 products, but due to 48 of them being registered with the co-occurrence of more than one compound, the total number of records was 235. The distribution of unauthorized PDE-5i through sub-groups of registered PDE-5i, including their analogues (Table 1), showed that the most common was sildenafil, which outnumbered tadalafil, vardenafil, and avanafil combined. Records related to sildenafil and tadalafil analogues and synthetic derivatives (61 in total) showed a rising trend over the years, due to producers’ attempts to avoid adulterant detection by available analytical methods. Over the observed period, the distribution of serious risk notifications between registered PDE-5i and their analogues went in favor of the former: out of 150 products, 69.3% contained one or more registered PDE-5i, while 30.7% were with synthetic analogues and/or their combinations with registered PDE-5i.

The safety profile of all four registered PDE-5i is comparable, with headache, flushing, nasal congestion, nausea, dyspepsia, vertigo, pain in the back and extremities, and myalgia being the most common adverse effects [28]. Moreover, it is believed that cases of sildenafil-related hepatotoxicity have been underestimated, as the symptoms of hepatic injury differ widely, and not all hepatotoxicity must be linked to sildenafil if its use is not transparent [29]. Due to the risk of symptomatic hypotension, the concomitant use of sildenafil and drugs used to treat benign prostatic hyperplasia or angina pectoris has been contraindicated [30]. Findings on the unconventional use of PDE-5i in healthy young adults between the age of 18 and 30 were quite alarming: recreational use was reported by 21.5% of respondents and more than half of them combined it with alcohol or other substances of abuse [31]. As nothing has been known about the pharmacokinetics and safety profile of unapproved analogues, they pose an extra safety concern [22]. Moreover, the safety and efficacy of combinations of two or more sub-types of PDE-5i have not been evaluated; hence, such mixtures should be avoided [30].

An analysis of the US Food and Drug Administration database in a period from 2007 to 2021 revealed 1068 unique supplements adulterated with pharmaceuticals, most commonly sexual enhancement and weight loss products. PDE-5i sub-types were commonly included in sexual enhancement supplements, with up to five representatives in a single product [32].

#### 3.2.2. Stimulants

Drugs that stimulate the central nervous system (CNS) activity fall under the umbrella term “stimulants” [33]. Stimulant thermogenic drugs are those that stimulate the CNS and potentially promote thermogenesis [34]. An extremely high number of people use stimulants for a wide range of indications, including, but not limited to, the improvement of performance, medical reasons, and recreational usage [33]. As this class of pharmaceuticals contains various amines with the same pharmacological effect, they are commonly referred to as phenylethylamines (PEA) [35].

Stimulants rank second among unauthorized pharmaceuticals in food supplements, closely following PDE-5i (Figure 3). The RASFF database revealed a rather versatile profile of natural and synthetic phenylethylamines and alkylamines (Table 1). Among natural ones, the most frequent was synephrine (53 records). The synthetic PEAs were lined in the following order: oxilofrine, β-methylphenylethylamine (BMPEA) and its derivative β-dimethylphenethylamine, isopropyloctopamine (deterenol), and amphetamine, whereas in two cases there was no official confirmation of the exact phenethylamine derivative. Alkylamine 1,3-dimethylamine (DMAA) was listed in 97 cases, 36 of which were in combination with at least one more stimulating compound, while its structural analogue 2-amino-6-methylheptane (DMHA) was much less represented. Among the serious risk notifications, cases of the individual occurrences of stimulants were recorded as follows: DMAA (41), synephrine (4), Ephedra (2), and BMPEA (1). The rest of the serious risk notifications (53.8%) were related to combinations of two or more stimulants: combinations containing DMAA (25); combinations containing synephrine (21), including two cases with octopamine and/or isopropyloctopamine; combinations containing DMAA, synephrine and/or among the other unauthorized substances oxilofrine (4); combinations containing BMPEA and oxilofrine (3); and combinations with ephedrine, norephedrine, or Ephedra (3).

The phenylethylamine alkaloid ephedrine from the *Ephedra* species is unquestionably the most well-known PEA with a long and extensive history of medical use. The effect of ephedra alkaloids has been described as similar to amphetamines, but less intense [36]. The number of reported adverse effects (hypertension, arrhythmias, myocardial infarction, convulsions, cerebrovascular insults, and death) increased along with the growing popularity of ephedrine in weight loss products and sports performance supplements [37]. Although ephedrine and its preparations have been prohibited for use in food supplements [38], a significant problem with ephedra alkaloids remains. It has been proven that both ephedrine and norephedrine can be easily chemically transformed into a far more potent and thus more dangerous amine, methamphetamine [39].

The era of “Ephedra free” supplements began with the outlawing of products containing ephedra alkaloids. Synephrine (*Citrus aurantium*, *Rutaceae*) was seen as a suitable and safe replacement in the treatment of obesity and the enhancement of athletic performance. According to the number of the RASFF notifications, synephrine was certainly the most prominent PEA illegally added to food supplements. Stohs et. [40] summarized the results of 23 human studies assessing the efficacy and safety of these products, and concluded that *p*-synephrine, alone or in combination, does not lead to significant side effects such as increase in heart rate or blood pressure. Nevertheless, with the increase in consumption, the number of reported side effects has increased significantly [40]. The proposed reason for this discrepancy was found in the fact that supplements for weight loss were almost always a combination of two or more active ingredients with a similar stimulating effect, so that their synergistic effect could easily occur (the combination of synephrine and caffeine was reported in 33 cases, 20 of which contained a high dose of caffeine). Taking *p*-synephrine and caffeine supplements causes an increase in cardiovascular stress at rest or during exercise, as well as changes in blood chemistry variables and mood perception [41,42]. A recent assessment of 30 case reports detailing the negative effects of supplements containing synephrine (the most listed were chest pain, syncope, dizziness, palpitations, myalgia, and headache), demonstrated the degree to which combinations of two or more stimulants should not be overlooked [43].

Oxilofrine, also known as methylsynephrine, is a sympathomimetic related to ephedrine. It is given orally as hydrochloride in the treatment of hypotensive states, and has also been used in antitussive preparations [44]. Like ephedrine, oxilofrine is on the World Anti-Doping Agency (WADA) list of prohibited substances in competition [45]. Oxilofrine-adulterated supplements gained a negative reputation after several athletes were removed from competition due to positive tests for oxilofrine, as well as the association of oxilofrine-containing supplements with serious adverse events such as vomiting, agitation, and cardiac arrest [46]. The findings of the studies investigating its presence in supplements showed over half of samples containing oxilofrine at concentrations far greater than those found in pharmaceuticals [46,47].

Various citrus species, primarily *Citrus limon* and *Rutaceae*, are plant sources of another PEA, octopamine. Because of its ability to elicit lipolysis, octopamine has been utilized in weight loss and sport performance products, despite poor evidence to support such use [48]. Due to its structural similarity to ephedrine and norephedrine, it would be expected to have similar adverse events accompanying adrenergic activity. However, even in human studies, when it was examined as a potential drug in the therapy of hypotension, it showed an almost insignificant increase in cardiac activity [48]. Octopamine is listed as a banned substance for professional athletes in competition by the WADA, although there is no reliable evidence of a stimulating effect or the existence of adverse events [45]. This seems exaggerated because it is based solely on structural similarity to other sympathomimetics [48].

Aegeline (*Aegle marmelos*, *Rutaceae*) is a PEA alkaloid which has antidiabetic and hypolipidemic effects that have been proven in vivo [49,50]. Following the consumption of food supplements under the trade names OxyElite Pro and VERSA-1, several cases of acute non-viral hepatitis have been recorded [51,52]. Although at the time no causal relationship between aegeline and hepatitis could be identified, a recent study has at least partly proved cytotoxicity and possible liver injuries caused by aegeline [53].

Despite the banning, BMPEA, a synthetic stimulant created as a potential substitute for amphetamine, has continuously been added to food supplements [54]. As a structural analogue of amphetamine, it is on the list of prohibited substances of the WADA, and analytical methods for its detection and differentiation from amphetamine in urine samples have been developed [45,55]. No human trials of BMPEA’s effectiveness or safety were ever conducted [56]. Nevertheless, there are documented cases of adverse events caused by supplements containing BMPEA [57], as well as a case report of hemorrhagic stroke probably caused by a combination of supplements containing BMPEA and exercise. This product had a combination of stimulants, including BMPEA, which was not listed, but specific dose of 290 mg cannot be considered unintentional contamination [58]. Even though there is evidence that BMPEA and similar analogs may show less potent effects on the cardiovascular system than amphetamines [59], combinations of BMPEA and comorbidities and/or stressful situations such as exercise may have fatal outcomes.

Isopropyloctopamine (deterenol) is a pharmaceutical beta-agonist and powerful hypotensive medication that does not cause mydriasis or miosis, which makes it suitable for glaucoma therapy [60]. This synthetic stimulant has a more pronounced lipolytic effect in vivo than its structural analogues synephrine and octopamine [61]. Its presence in weight loss, pre-workout, and fat-burning food supplements has been well documented [48,62,63], which is particularly alarming due to the prevalence of serious adverse events, primarily related to increased cardiac activity [57]. A case report from 2016 revealed heart problems leading to hospitalization and finally death, following patient overdose with a supplement containing multiple illegal stimulants (yohimbe alkaloids, synephrine, oxilofrine, caffeine, DMAA, theophylline, and BMPEA), but the cardiotoxicity could only be attributed to isopropyloctopamine [57,64]. The records in the RASFF database corroborate the practice of stimulant mixing: two of three showed combinations of isopropyloctopamine with yohimbine and BMPEA or with synephrine and high-dose caffeine.

Amphetamine (α-methylphenylethylamine) is a significant anorexic and central stimulant. Its oral administration is followed by tachycardia, hypertension, and behavioral changes, such as a sense of enhanced self-confidence and energy, but after a while, these are replaced by nervousness, depression, and lethargy [65]. Due to the substance’s high potential for abuse for recreational purposes, only narcolepsy and attention deficit/hyperactivity disorder (ADHD) have currently been approved as indications for medical use of amphetamine [66,67]. In addition to the predictable and frequent neurological side effects, several cases have reported the development of acute myocardial infarction due to the abuse of amphetamines in supplements mainly advertised for weight loss [68].

Synthetic stimulant DMAA was developed to replace amphetamine in over-the-counter (OTC) decongestants, but was relatively quickly withdrawn [69]. However, it gained more popularity as a bioactive compound in weight-loss and performance-enhancing products, as well as a recreational drug, despite its status as a doping agent and concerns about its safety [45,52,70]. It is known that DMAA affects bronchodilation, cardiovascular activity, and blood pressure [71]. Maintaining the position that products containing DMAA are adulterated and recall from the market has not prevented an increase in the reports of adverse events over the years. Cases of cerebral hemorrhage, cardiac arrests with fatal outcomes, cardiac failure, and heat stroke were reported following the recreational use of DMAA or DMAA contained in food supplements, in combination with exercising [70,72,73]. As has been mentioned, cocktails of stimulants are almost mandatory in food supplements—stimulants were the most numerous causes of multiple non-compliances recorded in the RASFF database over the observed period. DMAA was not an exception, and its combination with at least one more stimulating compound was recorded in around one third of DMAA-related notifications.

After the recall of DMAA, manufacturers, as has happened many times before, started using its structural analogues, such as DMHA (octodrine). With almost the same history in medicine as DMAA, DMHA was developed as a prescription drug and then withdrawn [69], with very limited data on its efficacy and safety in humans. In humans, the sympathomimetic effects of octodrine have been demonstrated to be bronchospasmolytic and hypertensive, whereas animal experiments have demonstrated the stimulation of the central nervous system, a local anesthetic effect, an increase in the pain threshold, and potential cardiotoxicity [74].

#### 3.2.3. Anorexics and Laxative

Based on the statistics of the World Health Organization, the prevalence of obesity has increased almost three-fold in just 40 years, with over 1.6 billion individuals being overweight and over 650 million being obese. According to the global burden of illness study, in 2017, over 4 million individuals died as a direct consequence of their obesity or overweight [75]. Moreover, modern society places a premium on being of a healthy weight and attractive physical appearance; nevertheless, many people struggle to achieve these goals on their own due to the time and effort required to implement the necessary nutritional, exercise, and lifestyle modifications. In these situations, food supplements that promise a fast fix could be perceived as an efficient solution [22].

The majority of 68 notifications caused by anorexics and laxatives were related to sibutramine (Table 1), 35 as a single compound and 10 in combination with other illegally added drugs, mostly phenolphthalein (7). It should be noted that 2,4-dinitrophenol (DNP) was frequently reported on RASFF as a substance offered for online sale (133), but only six notifications with an explicit link to food supplements were included in the current overview. Serious risk notifications (48.5% of all records) were mostly recorded in cases of the individual occurrence of sibutramine (16), DNP (6), phenolphthalein (2), and N-didesmethyl sibutramine (2), while the remaining ones were submitted in cases of combinations of anorexics and laxatives: sibutramine and phenolphthalein (4) and sibutramine and N-didesmethyl sibutramine (1), and 2 cases of sibutramine and sildenafil.

The adulteration of slimming preparations with synthetic compounds has become a major health problem, because unlike PDE-5i, which still in use in prescription drugs, laxatives and anorexics detected as adulterants in supplements are drugs banned due to their adverse effects. The most common representatives are sibutramine and its active metabolite N-didesmethylsibutramine, phenolphthalein, and DNP. Pharmaceuticals containing sibutramine had been previously authorized in the EU since 1999, under different tradenames, for the treatment of obesity and overweight associated with additional risk factors. Following the results of the Sibutramine Cardiovascular OUTcomes (SCOUT) study, proving the association between sibutramine and cardiovascular disorders, the European Medicines Agency recommended withdrawal, suspension, or changes to the marketing authorization, which happened in 2010 [76]. Additionally, several reports and case studies have been published on the neuropsychiatric side effects of sibutramine, which may represent a genuine danger under conditions of increased stress or in patients who have a hereditary susceptibility for neuropsychiatric disorders [77]. Following sibutramine withdrawal from the market, its analogues were developed at a rapid pace. N-didesmethyl sibutramine, one of its active metabolites, was nevertheless “frequently” included in weight-loss food supplements, typically at levels much greater than those classified as therapeutic (which has an allowed daily dose of 15 mg) [78].

Another drug that is very often on the list of notifications related to counterfeit supplements is phenolphthalein, a laxative that has been approved for use in several over-the-counter medications in the past [44]. Following the American National Toxicology Program assessment showing indications of its mutagenic and carcinogenic potential, phenolphthalein was withdrawn many years ago [79].

DNP was one of the first medications utilized for the treatment of obesity [80]. However, rapid weight reduction achieved with DNP comes with an unacceptable risk of serious side effects. DNP-containing supplements are mostly advertised online as weight loss/slimming assistance under various names [81]. The toxicity of DNP is characterized by a complex clinical condition that includes hyperthermia, tachycardia, diaphoresis, and tachypnoea, and finally leads to death. Grundlingh et al. [81] reported 62 fatalities linked to DNP in medical literature.

Tendencies in illegal food supplements specifically for weight loss were previously investigated on the basis of RASFF reports in the time period of 1988–2019 [21]. Among more than 2500 food supplements with identified quality problems, 319 were marketed to facilitate weight loss and 202 of these contained unauthorized synthetic drugs. The most frequent adulterant was DNP (113 reports), followed by sibutramine (69). The US Food and Drug Administration database also emphasized sibutramine as the most included adulterant in weight loss products over the period 2007–2021, along with sibutramine analogues, phenolphthalein, and fluoxetine [32].

#### 3.2.4. Nootropic Drugs

Among vincamine derivatives, vinpocetine was the one occurring most frequently, followed by vincamine and vinburnine (Table 1). There was only one serious alert (Figure 5), but that was mostly related to the period of records (until 2012).

Although vinpocetine has been used clinically in the treatment of cerebrovascular disorders due to its neuroprotective and nootropic effects [82], in the summary of the characteristics of the drug Cavinton^®^, only occasional, rare, and very rare side effects of mild intensity, dizziness, headache, and hypotension were described. When it is used for therapeutic purposes, it has a good safety profile; a summary of clinical studies showed no serious adverse effects [83]. However, vincamine-adulterated supplements promoted to enhance memory and mental attention indirectly endanger the consumer’s health by making it impossible to establish the correct diagnosis and treatment of a patient with neurological disorders and by unknowingly combining them with contraindicated medicines, such as drugs that prolong the QT interval [63,82]. The biggest concern is that they may contain multiple unauthorized substances and/or unapproved drugs in typical pharmacological doses [63]. Only one serious alert recorded for nootropic drug adulterants could be attributed in part to their afore-described pharmacology and safety profile. It was related to a supplement containing vinpocetine in a concentration significantly higher than the pharmacologically active dose (2.5 mg/kg).

#### 3.2.5. Anabolic Androgenic Steroids (AAS)

Supplements adulteration with AAS, also called prohormones, has been demonstrated numerous times [84,85,86], although the only justified use of testosterone and AAS is in hormone replacement therapy [87]. The majority of 12 RASFF notifications related to these compounds were submitted as serious risk (75%), 4 of them being individual occurrences of AAS, androstenedione (3), and 4,9-estradien-3,17-dione (1), while remaining ones were related to combinations of androstenedione and progesterone (2), androstenedione and delta-9-tetrahydrocannabinol (1), methasterone and DMAA (1), and 4,6-androstadien-17-ol-3-one, 4,6-androstadien-3,17-dione, 5-androsten-17-ol-3-one, testosterone, stanozolol and DMAA (1).

Since AAS are generated from testosterone and its associated precursors [88], when they are chronically abused, their side effects become permanent and gender-specific—gynecomastia, hirsutism, hypertrophy, prostate cancer, hepatotoxicity, kidney damage, increased cardiac activity, and cardiac death [89,90,91]. There is a far higher danger to public health from the synthetic or designer steroids (such as recorded for methasterone, 4,6-androstadien-17-ol-3-one, 4,6-androstadien-3,17-dione, 5-androsten-17-ol-3-one, 4,9-estradien-3,17-dione), because almost nothing is known about their pharmacology and the effects of long-term exposure. Clinical studies have shown that dehydroepiandrosterone (DHEA) supplements, often at a dosage of 25 mg, have been beneficial in treating illnesses, including adrenal insufficiency, and providing hormone replacement therapy in postmenopausal women [92]. Nevertheless, the risks of using DHEA supplements are related to androgenic side effects, hirsutism, and acne, especially in cases of contraindicated use in patients with breast cancer or elderly women with heart problems [93]. In addition to serious health-related risks caused by intentional adulteration with considerably higher than the normal therapeutic doses, there is a major risk for a high-level-competition athlete to have a positive drug test result, even after exposure to very low doses, as even low doses are sufficient to give a positive result on a doping test [94], since all AAS are banned both in- and outside of competition [45].

#### 3.2.6. Cannabinoids

Notifications related to supplements adulterated with Δ-9-tetrahydrocannabinol (THC; unauthorized substances) and cannabidiol (CBD; unauthorized novel food ingredient) start to be submitted from 2014 onwards. However, less than half of the notifications (45.8%) were submitted with a serious risk decision, six in case of adulteration with a combination of THC and CBD, four in cases of individual occurrence of THC, and one notification of THC and androstenedione, all classified as alerts.

Numerous European countries restricted the prescribing of medical products containing the main pharmacologically active components of *Cannabis sativa*, *Cannabidaceae*, THC and CBD, for indications other than pain management, including cancer treatment, AIDS, multiple sclerosis, and rare conditions such as Lennox–Gastaut, Tourette’s, or Dravet syndrome [95]. Nevertheless, even for these indications, their clinical utility must be validated by additional high-quality clinical trials. To date, cannabinoids’ use has been linked to statistically significant but modest improvements in pain relief and physical functioning, as well as an increased risk of regurgitation (high quality evidence) [96], and sleepiness, vertigo, and mental issues were reported with all cannabis-based medications (evidence of low to moderate quality) [97]. Another unknown that needs to be investigated when dealing with cannabis-based medical products is possibility of drug–drug interactions. Although data on interactions are obscure, due to the way CBD is metabolized it interacts with antiepileptics, benzodiazepines, opioid analgesics, and tricyclic antidepressants [98], and a small number of cases indicate the interaction of THC with warfarin [99].

Polish researchers conducted an interesting study connecting food supplements and medicinal products through the phenomenon of borderline ingredients, i.e., ingredients applied in both supplements and medicines, including cannabinoids. They compared mostly questioned supplements from their national register of functional products with data from registers of pharmaceutical substances and from the RASFF (limited to supplements indicating Poland in the subject of notification or as a notifying country within the time period 2020–2022). The analysis revealed the dishonest market practice of producers and distributors of non-conforming supplements, as well as a tendency to re-notifications of the questioned products with an aim to keep them on the market. Thus, 10 supplements containing CBD were re-notified within time periods ranging from 15 to 145 days, a further 10 with Cannabis sativa from 80 to 664 days, and 3 with phytocannabinoids from 12 to 35 days [23].

#### 3.2.7. Unclassified Pharmaceuticals

5-hydroxytryptophan (5-HTP), betaine, ciprofloxacin, and zopiclone are pharmaceuticals identified as adulterants in food supplements that cannot be classified in any of the previously listed categories. Three out of seven notifications related to 5-hydroxytryptophan (5-HTP) were due to its combined presence with melatonin, two of them submitted as serious risk notifications (one alert, one information). Betaine was recorded as an unauthorized novel food ingredient, but all records were until 2019 and not one was a serious alert or border rejection.

As a precursor of serotonin, 5-HTP has been used in the treatment of depression and other neurological disorders, with greater success as an adjunctive therapy [100]. Increases in serotonin levels, although helpful in the treatment of neurological diseases, may have a potentially lethal side effect known as serotonin syndrome. When used in conjunction with other medications that share its mechanism of action, the chance of acquiring this syndrome increases [101,102]. Betaine, known under the tradename Cystadane, was classified as an unauthorized novel food ingredient until 2019, when its status changed [103]. Unintended adulteration due to cross-contamination and/or low-quality production is presumably the reason for the isolated occurrences of ciprofloxacin and zopiclone.

### 3.3. Overall Considerations

In the case of pharmaceutical substances, it is of pivotal importance to consider RASFF records not only as numbers and technical descriptors, but also in the context of safety profiles and side effects associated with specific pharmaceuticals and their combinations. One of the crucial aspects is that consumers of food supplements are seeking a specific desirable effect, most often being unaware of the presence of unauthorized pharmaceuticals and consequent health risks. The lack of disclosure of pharmaceuticals in food supplements, circumventing clinician oversight of drug use, and the use of banned pharmaceuticals or never-studied combinations of pharmaceuticals are crucial health risks for consumers [32]. The use of food supplements is common among patients, most of whom tend not to disclose supplement use to the medical staff. Thus, in the case of adulterated products, in addition to side effects related to a specific pharmaceutical substance itself, risks include potential and actual interactions with prescription drugs [104].

Although the analysis of RASFF data presented in the current study supports their efficiency and usefulness for keeping food supplement consumers informed and safer, several limitations of the data publicly available on the RASFF portal should be noted. Firstly, due to the absence of the identification of hazardous products with “hidden” ingredients, consumers are not efficiently alerted. Conversely, the US FDA “Tainted Products Marketed as Dietary Supplements” web portal provides product-specific details, including the product and manufacturer name, product category, and lot number(s) that were associated with adulteration, as well as “hidden” pharmaceutical ingredients [105]. The US FDA also provides information on supplement categories likely to be adulterated (“Medication Health Fraud” general guidelines) [106]. Furthermore, in 2019, the US FDA unveiled the “Dietary Supplement Ingredient Advisory List”, identifying ingredients that do not appear to be lawfully marketed in food supplements and need to be further evaluated in terms of their safety [105]. Thus, manufacturers/consumers are provided with information about ingredients/products that they may wish to avoid adding into their products/purchasing. Importantly, the US FDA has the authority to apply an arsenal of enforcement tools, including product recalls/seizures, warning letters (published on the FDA website), temporary restraining orders, consent decrees, injunctions, civil and criminal prosecution, and fines [3]. Health Canada maintains a database of natural health products licensed for sale in Canada, as well as detailed information on recalls and safety alerts with the product name, manufacturer details, and even images of non-authorized products. Post-marketing surveillance includes the voluntary reporting of adverse events by healthcare providers and consumers through the Canadian Vigilance system. The development of the Medeffect Program should provide centralized access to relevant safety information, not only for drugs and medical devices, but also for food supplements [11]. The differences between the systems could be exploited for their improvement—approaches and solutions that have been proven to be effective in one system could be transferred to another. Returning to the RASFF, it does not provide information on whether the products are marketed for the same indication as the unauthorized pharmaceutical detected in the product, as it would be of particular concern if the pharmaceutical is indicated for addressing a different condition. Such information would be helpful not only for consumers but also for monitoring authorities deciding what to test in a product, as considering that unauthorized pharmaceuticals were not identified on the product label and laboratory testing is of crucial importance. Information on the levels of pharmaceuticals found in the products is rarely available (mass per kg/per dosage unit/per item or only indication “detected”, see Table 2), and never accompanied with dosage instructions (along with unknown identity of the product), thus precluding the assessment of whether the consumed doses may exceed pharmaceutical doses and/or cause adverse health effects, although risk decision provided by RASFF does indicate whether the risk is serious or not.

There are limited guidelines and no consensus methods for determining the human health risk associated with unauthorized pharmaceuticals in food supplements. However, a tier-based framework incorporating typical lines of evidence has recently been developed. The framework outlines the hazard identification and decision to test for pharmaceutical substances in products, based on the assessment of the probability of adulteration (all high-risk-category products should be subjected to chemical analysis) and manufacturer production standards (the lack of specific manufacturing certifications or product origin could be used for prioritization for screening). For products with detectable levels of pharmaceuticals (tested by certified laboratories with proper protocols), several proactive approaches have been proposed [107]. The first is the use of fraction of the therapeutic dose, in terms of comparison of the pharmacological potency of the pharmaceutical substance determined in the food supplement to the smallest therapeutic dosage for registered drug (taking into account routes of exposure), representing the lowest observed effect level (LOEL) for pharmacology, coupled with an adequate uncertainty factor. Products containing pharmaceuticals that surpass the lowest therapeutic doses are considered adverse to the general public. When data on the therapeutic dose of a pharmaceutical substance are unavailable, the alternative could be the use of the thresholds of toxicological concern (TTC), providing that the compound is not genotoxic and/or carcinogenic, a reproductive and developmental toxin, a hormone, or a sensitizer. For compounds containing multiple pharmaceuticals or when multiple class-specific TTC levels are of relevance, the lowest TTC should be adopted for risk characterization. Safety concerns are triggered for products with drug levels above the TTC. For any product that is considered to pose a potential health risk, the derivation of a specific health-based exposure limit (HBEL) is recommended, considering both clinical and nonclinical data and the pharmacokinetics and pharmacodynamics of the drug. The product causing exposure above the HBEL would be considered to pose an appreciable health risk [107].

It should be noted that even when an adulterated product has been identified and restrictive measures have been taken, this still does not prevent unscrupulous manufacturers from re-marketing the same product with the same or similar illegally added ingredients. Such a situation was documented in a summary of the trends across adulterated food supplements associated with a US FDA warning released between 2007 and 2016, showing that 28 of 776 products adulterated with pharmaceutical ingredients were the subjects of multiple warnings issued more than 6 months apart [108].

Caution should be exercised in using the RASFF database either as a predictive tool or for trend analysis, due to the impact of iterative changes in food law on the frequency of regulatory sampling both for border and inland regulatory control [109]. However, in cases of unauthorized pharmaceuticals, the RASFF findings are probably more affected by the very rapid development of analytical methods and an increase in the capacity of testing laboratories. A multi-analyte screening method for the rapid detection of pharmaceutically active substances in food supplements at realistic adulteration levels has been under development. Although the low specificity of such methods could be seen as their flaw, it would actually benefit the detection of new analogues of the target pharmaceuticals. In order to be used by inspectors for screening at customs or sale points, these methods have to enable quick and easy in situ analyses. A method based on a portable SERS (Surface-Enhanced Raman Scattering) analyzer was successful in the qualitative identification of eleven out of twenty-three illicit adulterants in weight-loss, energy boosting, and sexual and sport performance enhancement supplements [110].

Finally, the need to detect and scrutinize the adverse health effects of food supplements strongly advocates for the creation of a coordinated European nutrivigilance system—important lessons for its development and effective functioning could be transferred from the International Nutrivigilance network [12] and other surveillance systems such as the MedWatch program/CAERS (FDA) in the US, Medsafe in New Zealand, and the Pharmacovigilance (EMA) in the EU [8]. 

## 4. Conclusions

The alarming number of RASFF notifications on the illegal use of pharmaceuticals in food supplements clearly shows that the existing regulations do not efficiently prevent irresponsible manufacturers from falsifying their products. The analysis of RASFF reports showed the level of seriousness when it comes to supplements falsified by pharmaceuticals (the majority were classified as serious risk); thus, a clear and targeted recommendation for action for the legislature and authorities are urgently needed. Considering that this is a problem globally, better communication worldwide on products of risk/emerging areas of risk is of pivotal importance. A need for the harmonization and reduction of the regulatory differences between producing and selling countries, as well as the creation of stricter legal regulations that will refer to the minimum safety requirements that manufacturers must meet before commercializing the product, must be recognized and acknowledged. Measures to decrease the level of concern should include repeated and more frequent inspection controls on the market. It should be noted that the consumers of supplements for weight loss, sports performance improvement, or memory improvement are the most exposed. The consequences for public health caused by adulterated supplements are one of the most pressing problems. Therefore, a harmonized nutrivigilance system should be considered as a tool to detect and scrutinize the adverse health effects of food supplements and reinforce the actions of policy makers in protecting and informing consumers in a timely manner.

## Figures and Tables

**Table 1:** Unauthorized pharmaceuticals identified in food supplements—number of notifications (N) (RASFF database, 2011–2022).

Unauthorized Substance	Trade-Name ^1^	Indications/Use	N
**Phosphodiesterase-5 inhibitors (PDE-i)**			
Sildenafil	Viagra	Erectile dysfunction, pulmonary arterial hypertension	99
-sildenafil thiono analogue			46
-sildenafil analogue			2
-dimethylsildenafil			7
-nor-acetildenafil			1
Tadalafil	Cialis	Erectile dysfunction	63
-desmethyl tadalafil			1
-nortadalafil			3
Vardenafil	Levitra	Erectile dysfunction	11
-pseudovardenafil			1
Avanafil	Spedra	Erectile dysfunction	1
**Stimulants**			
*Natural phenylethylamines (PEA)*			
Ephedrine, Norephedrine, Ephedra	Efedrosan, Neo Rhinovit, Rhinolex, Kondon’s Nasal	Sympathomimetic relief in nasal congestion, bronchodilatation in asthma therapy	5
Synephrine	Neo-Synephrine, Ocu-Phrin, Visadron	Decongestant, mydriatic, vasopressor agent in surgical procedures	53
Octopamine	Norphen	Hypotension therapy and cardiotonic	4
Aegeline	Oxyelite Pro, VERSA-1	Designer stimulant	1
*Synthetic phenylethylamines (PEA)*			
Oxilofrine	Carnigen	Hypotension therapy, antitussive preparations	8
β-Methylphenylethylamine (BMPEA)	Dexaprine, Jacket Power, MM Sports	Designer stimulant	6
Isopropyloctopamine	Dexaprine	Designer stimulant	3
α-Methylphenylethylamine (amphetamine)	Adderall	Therapy of ADHD and other neurological disorders	1
*Alkylamines*			
1,3-Dimethylamine (DMAA)	OxyElite Pro, Jack3d, Napalm	Designer stimulant	97
2-Amino-6-methylheptane (DMHA, octodrine)	Ambredin	Designer stimulant	6
**Anorexics and laxatives**			
Sibutramine	Reductil, Reduxade, Zelium	Obesity	45
N-Didesmethyl sibutramine	-	-	7
Phenolphthalein	Alin, Laksafenol, Fam-Lax, Agoral	Constipation	18
2,4-Dinitrophenol (DNP)	-	Obesity	6
**Nootropic drugs**			
Vinpocetine	Cavinton	Cerebrovascular disorders, mental function impairment	18
Vincamine	Cerebroxine, Vincapan		3
Vinburnine	Cervoxan		3
**Anabolic androgenic steroids (AAS)**			
Androstenedione	Metharmon-F	Hormone replacement therapy	6
Stanozolol	Winstrol	Hereditary angioedema, vascular disorders	2
Dehydroepiandrosterone (DHEA)	Biosteron	Hormone replacement therapy	2
Testosterone	Andriol, Androgel	Testosterone replacement therapy	1
Methasterone	-	Designer ASS	1
4,6-Androstadien-17-ol-3-one	-	Designer AAS	1
4,6-Androstadien-3,17-dione	-	Designer AAS	1
5-Androsten-17-ol-3-one	-	Designer AAS	1
4,9-Estradien-3,17-dione	-	Designer AAS	1
**Cannabinoids**			
Delta-9-tetrahydrocannabinol (THC)	Marinol, Cesamet	Cancer treatment, AIDS, multiple sclerosis	23
Cannabidiol (CBD)	Epydiolex	Lennox–Gastaut syndrome, Dravet syndrome	14
THC + CBD	Sativex	Multiple sclerosis	13
**Others (Unclassified pharmaceuticals)**			
5-HTP	Levotonine, Trip-OH	Depression, neurological disorders	7
Betaine	Cystadane	Homocystinuria	7
Zopiclone	Zimovane	Hypnotic, Insomnia	1
Ciprofloxacin	Ciproxin, Ciloxan, Cetraxal	Antibiotic	1

^1^ Registered trade names in EU and/or USA according *Martindale: The Complete Drug Reference* and Drugs@FDA database or relevant literature in case of designer stimulants.

**Table 2:** Unauthorized pharmaceuticals identified in food supplements—summary of determined concentrations (RASFF database, 2011–2022).

Unauthorized Substance	Number of Samples			Concentration								Dosage
	Conc. Not Available/Quantified	“Suspected”	“Detected”	mg/Item	mg/cps	mg/dosage	mg/kg	mg/kg Dry Matter	mg/100 mL	mg	%	mg/Day
**Phosphodiesterase-5 inhibitors**												
Sildenafil	45/44		10	>7–146.3	0.318–104.7	36.2–58.34	2.6–244,000	72,650–349,820	2.8			
Sildenafil thiono analogue	34/11		1	>20–114.7	0.07–140		347				2.25–14.7	
Sildenafil analogue	0/0		2									
Dimethylsildenafil	4/3			67–78			89					
Nor-acetildenafil	0/1			106								
Tadalafil	26/30		7	0.023–20			4.6–147,000	80.6		14–49.2		
Desmethyl tadalafil	0/0		1									
Nortadalafil	0/1						0.107–120,000			34		
Vardenafil	9/3	1		> 5								
Pseudovardenafil	0/0	1										
Avanafil	1/0											
**Stimulants**												
DMAA	72/25			12.5–270	36.5	60	2.95–55,300					
DMHA	6/0											
Synephrine	34/19			23.23–44.1			75–21,220		646			75–76
Ephedrine, Norephedrine and Ephedra	5/0											
Octopamine	1/3			200	46.1		107,500					
Aegaline	1/0											
Oxilofrine	4/4						4300–45,740					
BMPEA	4/2						9800–10,800					
Isopropyloctopamine	2/1						7000					
Amphetamine	1/0											
**Anorexics and laxatives**												
Sibutramine	16/29			0.053–17.4	16.2	15.9	63–22,830					
N-Didesmethyl sibutramine	1/6						0.531–3020					
Phenolphthalein	4/14			5.1			3.8–6093	950				
2,4-Dintrophenol	5/1						11					
**Nootropic drugs**												
Vinpocetine	16/1		1				2.5					
Vincamine	3/0											
Vinburnine	3/0											
**Anabolic androgenic steroids**												
Androstendione	1/5						0.103–5.57					
Stanozolol	0/2						1.7–11					
DHEA	1/1						20					
Testosterone	0/1						2403					
Methasterone	0/1				7.6							
4,6-Androstadien-17-ol-3-one	0/1						5142					
4,6-Androstadien-3,17-dione	0/1						199					
5-Androsten-17-ol-3-one	0/1						6327					
4,9-Estradien-3,17-dione	0/1						28,606					
**Cannabinoids**												
THC	0/23						0.101–2980	324				
CBD	8/6			5			12,000–32,100					
**Other**												
5-HTP	5/2			100						30		
Betaine	7/0											
Zopiclone	1/0											
Ciprofloxacin	1/0											

## Data Availability

The datasets generated for this study are available on request to the corresponding author.
